# Identification of the minimum region of *Bordetella pertussis* Vag8 required for interaction with C1 inhibitor

**DOI:** 10.1111/1348-0421.12799

**Published:** 2020-07-02

**Authors:** Naoki Onoda, Yukihiro Hiramatsu, Shihono Teruya, Koichiro Suzuki, Yasuhiko Horiguchi

**Affiliations:** ^1^ Department of Molecular Bacteriology, Research Institute for Microbial Diseases Osaka University Suita Osaka Japan; ^2^ The Research Foundation for Microbial Diseases of Osaka University (BIKEN) Suita Osaka Japan

**Keywords:** *Bordetella pertussis*, C1 inhibitor, Vag8

## Abstract

An autotransporter of *Bordetella pertussis*, virulence‐associated gene 8 (Vag8), binds and inactivates the complement regulator, C1 inhibitor (C1‐Inh), and plays a role in evasion of the complement system. However, the molecular interaction between Vag8 and C1‐Inh remains unclear. Here, we localized the minimum region of Vag8 required for interaction with C1‐Inh by examining the differently truncated Vag8 derivatives for the ability to bind and inactivate C1‐Inh. The truncated Vag8 containing amino‐acid residues 102–548, but not 102–479 and 202–648, showed the full activity of intact Vag8, suggesting that the separate 102–202 and 548–648 amino‐acid regions of Vag8 mediate the interaction with C1‐Inh.

**Abbreviations**
C1‐Inh: C1 inhibitor
D‐PBS: Dulbecco's‐modified phosphate buffered saline
OD_450_: optical density at 450 nm
PK: plasma kallikrein
RCL: reactive center loop
Vag8: virulence‐associated gene 8

*Bordetella pertussis* causes pertussis (whooping cough), a contagious respiratory disease that has recently seen a resurgence despite high vaccination coverage, which has prompted attempts to improve current pertussis vaccines.^1^, ^2^ A number of groups have attempted to identify novel bacterial components capable of conferring efficient immunity against *B. pertussis* infection, and virulence‐associated gene 8 (Vag8) has been proposed as a possible candidate.^3^, ^4^ Vag8 is an autotransporter, which is autonomously secreted by an intramolecular system consisting of passenger and translocator domains. After passing through the inner membrane by a canonical secretion system, the passenger domain is translocated across the outer membrane by the translocator domain. The passenger domain of Vag8 is cleaved and liberated into the extracellular milieu.^5^ The liberated Vag8 binds and inactivates the complement regulating factor, C1 inhibitor (C1‐Inh).^6^ C1‐Inh inhibits various types of plasma serine proteases, including plasma kallikrein (PK) and factor XIIa in the contact system, and the proteases of C1 and MASP complexes in the complement system.^7^ The inactivation of C1‐Inh by Vag8 leads to unregulated activation of the proteases, resulting in PK‐bradykinin‐mediated inflammation via the contact system and depletion of the complement components.^5^, ^8^ Therefore, Vag8 is considered to contribute to the pathogenicity and virulence of *B. pertussis*. However, the nature of the molecular interaction between Vag8 and C1‐Inh remains unclear. Here, we attempted to localize the minimum region of Vag8 required for interaction with C1‐Inh by examining different truncated Vag8 derivatives for the ability to bind and inactivate C1‐Inh. Our results suggested that at least two separate regions of Vag8 are necessary for interaction with C1‐Inh.

We generated nine truncated Vag8 derivatives covering the passenger domain along with the wild‐type, Vag8_WT_ (Figure 1a). DNA fragments encoding WT and truncated derivatives of Vag8 were amplified from *B. pertussis* type strain 18323 or vaccine strain Tohama using appropriate primers (Table 1), and cloned into pCold II‐HAT.^3^ The expression of each Vag8 derivative in *Escherichia coli* BL21 (DE3) or DH5α harboring each expression vector was induced with 1 mM isopropyl β‐d‐1‐thiogalactopyranoside. The collected bacteria were lysed in 50 mM sodium phosphate buffer, pH 8.0, containing 300 mM NaCl (buffer A) by sonication. After centrifugation, the pellets were dissolved in buffer A containing 8 M urea and dialyzed against buffer A. The resultant samples were centrifuged, and the supernatants were independently applied to a column of HIS‐Select Nickel Affinity Gel (Sigma‐Aldrich) equilibrated with buffer A. After non‐absorbed substances had been washed out of the column with 50 mM sodium phosphate buffer, pH 6.3, containing 300 mM NaCl, the recombinant Vag8 proteins were eluted with 50 mM sodium phosphate buffer, pH 4.5, containing 300 mM NaCl, and 300 mM imidazole. Imidazole in the Vag8 fraction was removed by dialysis against buffer A. Protein concentrations were determined using a micro BCA protein assay kit (Thermo Fisher Scientific). The recombinant proteins were subjected to SDS‐PAGE followed by immunoblotting using anti‐HAT‐tag antibody (GenScript) and HRP‐conjugated anti‐rabbit IgG (Jackson ImmunoResearch). Target proteins were visualized with Immobilon Western Chemiluminescent HRP substrate (Merck Millipore). The mobilities of the recombinant Vag8 proteins on SDS‐PAGE followed by immunoblotting corresponded to those estimated from their respective molecular masses (Figure 1b).

**Figure 1:**
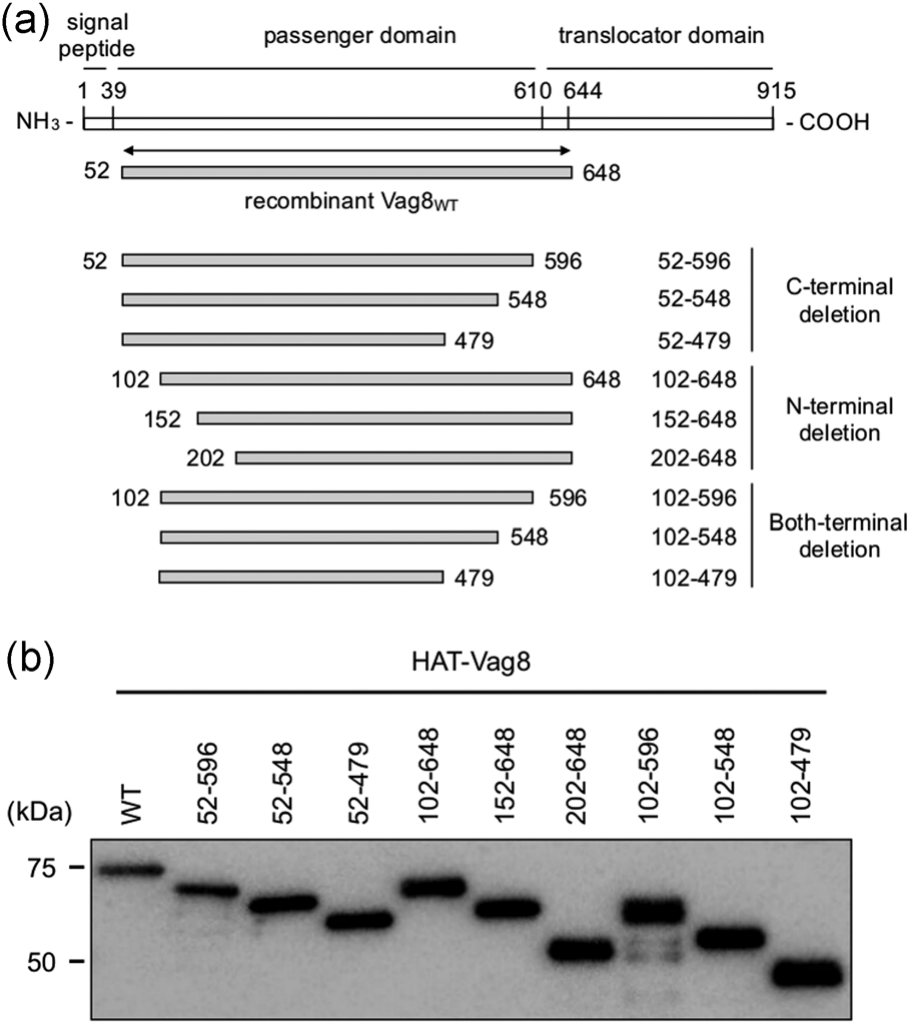
Vag8 and its derivatives used in this study. (a) Schematic representations of Vag8 and each recombinant protein are shown. Vag8_WT_ and truncated derivatives of Vag8 are listed with their names and corresponding amino‐acid positions. Each recombinant protein had a HAT‐tag at the N‐terminus. (b) Immunoblotting of the recombinant Vag8_WT_ and truncated derivatives from *B. pertussis* 18323

**Table 1:** Primers used in this study

Primer	Sequence (5′‐3′)	Vag8 derivatives
vag8‐*Xho*I	CACAACAAGCTCGAGTTTTCTGGGCGATGTC	Vag8_WT_
vag8‐*Eco*RI	AAGCTTGAATTCCTACCCGATCGAATGCAG	Vag8_WT_
pVag1	ATGCCCACAACAAGCTCGAGTTTCTGG	Vag8_52‐596, 52‐548, 52‐479_
pVag2	CTGCAGGTCGACAAGCTCTACCGGGAATCGCCCGTCACG	Vag8_52‐479, 102‐479_
pVag6	ACTGCAGGTCGACAAGCTTGAATTCC	Vag8_102‐648, 152‐648, 202‐648_
pVag9	TGCCCACAACAAGCTCGAGCCGGACAACACCGCGCT	Vag8_102‐648, 102‐596, 102‐548, 102‐479_
pVag10	TGCCCACAACAAGCTCGAGGGCGATGACAGTTTCGCCCT	Vag8_152‐648_
pVag11	TGCCCACAACAAGCTCGAGGGGGCAGGCGTTTCCGCGA	Vag8_202‐648_
pVag14	CTGCAGGTCGACAAGCTCTACGTGTTGTTGACCACCAGCA	Vag8_52‐548, 102‐548_
pVag15	CTGCAGGTCGACAAGCTCTAGGCCTGCGCCTCGC	Vag8_52‐596, 102‐596_

The ability of each recombinant Vag8 to bind C1‐Inh was quantified by enzyme‐linked immunosorbent assay (ELISA)‐based binding assay. Wells of 96‐well microplates (ELISA Plate H; Sumitomo Bakelite) were coated with 0.1 mL of 10 nM C1‐Inh (Complement C1 Inhibitor, Human; Calbiochem) at 4°C overnight, and then blocked with Dulbecco's‐modified phosphate buffered saline (D‐PBS) containing 10% skim milk at 37°C for 1 hr. Recombinant Vag8 proteins at the indicated concentrations were added to the wells and allowed to react at 37°C for 1 hr. The Vag8 proteins bound to C1‐Inh were probed with a combination of anti‐HAT‐tag antibody, HRP‐conjugated anti‐rabbit IgG, and TMBZ (Dojindo Laboratories). Each antibody reaction was carried out at 37°C for 1 hr. The substrate reaction was carried out at room temperature for 30 min and stopped by the addition of 1 N H_2_SO_4_. After each step before the substrate reaction, the wells were washed with D‐PBS containing 0.05% Tween‐20. The optical density at 450 nm (OD_450_) of each well was read with a multi‐detection microplate reader (Powerscan HT; BioTek). The interactions between C1‐Inh and the Vag8 derivatives were also examined based on the inhibitory effect of C1‐Inh on the protease activity of PK according to the procedure described previously with minor modifications.^8^, ^9^ Briefly, recombinant Vag8 proteins (1 µM) were incubated with 50 nM C1‐Inh, and subsequently with 2 nM PK (Human Kallikrein; Enzyme Research Laboratories) at 37°C for 1 hr each. After incubation, the solution was mixed with the chromogenic substrate (Chromogenix S‐2302, 0.5 mM; Diapharma) in HEPES‐NaHCO_3_ buffer,^9^ and incubated at 37°C for 1 hr. The OD_405_ value of each well, determined using a multi‐detection microplate reader, was used as an indicator of the protease activity of PK. In PK‐C1‐Inh complex formation assay, recombinant Vag8 proteins (3 µM) were preincubated with 100 nM C1‐Inh at 37°C for 1 hr in 27 mM HEPES buffer, pH 7.4, containing 191 mM NaCl, and 0.68 mM EDTA.^10^ The resultant mixture was mixed with 100 nM PK, incubated at 37°C for 1 hr, and subjected to SDS‐PAGE followed by immunoblotting using anti‐C1‐Inh antibody (Abcam) and HRP‐conjugated anti‐rabbit IgG as described above.

We first confirmed that Vag8 of *B. pertussis* strains 18323 and Tohama (Vag8_18323_ and Vag8_Tohama_, respectively) (Figure 1a), which share 99.9% identify at the amino‐acid sequence level, had comparable C1‐Inh binding abilities (Figure 1a), and used Vag8_18323_ and its truncated derivatives as test materials for further experiments. As shown in Figure 2, among the C‐terminal deletion mutants, Vag8_52‐596_ and Vag8_52‐548_ showed C1‐Inh binding capability similar to Vag8_WT_, while the binding of Vag8_52‐479_ was markedly reduced (Figure 2b, left panel). The N‐terminally deleted derivatives showed decreased binding with extension of the deletion (Figure 2b, center panel). Derivatives with deletion at both termini, that is, Vag8_102‐596_ and Vag8_102‐548_, showed similar C1‐Inh binding capability to Vag8_WT_, while the binding of Vag8_102‐479_ was markedly reduced (Figure 2b, right panel). Taken together, these observations indicated that the region from amino‐acid residue 102–548 is required for binding to C1‐Inh. Next, we examined the interactions between C1‐Inh and the Vag8 derivatives. Vag8_52‐596_, Vag8_52‐548_, Vag8_102‐648_, Vag8_102‐596_, and Vag8_102‐548_ inhibited the C1‐Inh activity to the same level as Vag8_WT_, while Vag8_52‐479_, Vag8_202‐648_, and Vag8_102‐479_, which scarcely bound to C1‐Inh, did not (Figure 2c). Vag8_152‐648_, which showed intermediate C1‐Inh binding ability, did not inhibit the function of C1‐Inh, suggesting that the domains binding and inactivating C1‐Inh are independently localized on the Vag8 molecule. In addition, we examined whether the truncated Vag8 derivatives could inhibit formation of the complex of PK and C1‐Inh by a mobility shift assay on SDS‐PAGE followed by immunoblotting. The truncated Vag8 derivatives carrying the minimum region for association with C1‐Inh inhibited covalent binding between PK and C1‐Inh, while the others did not (Figure 2d).

**Figure 2:**
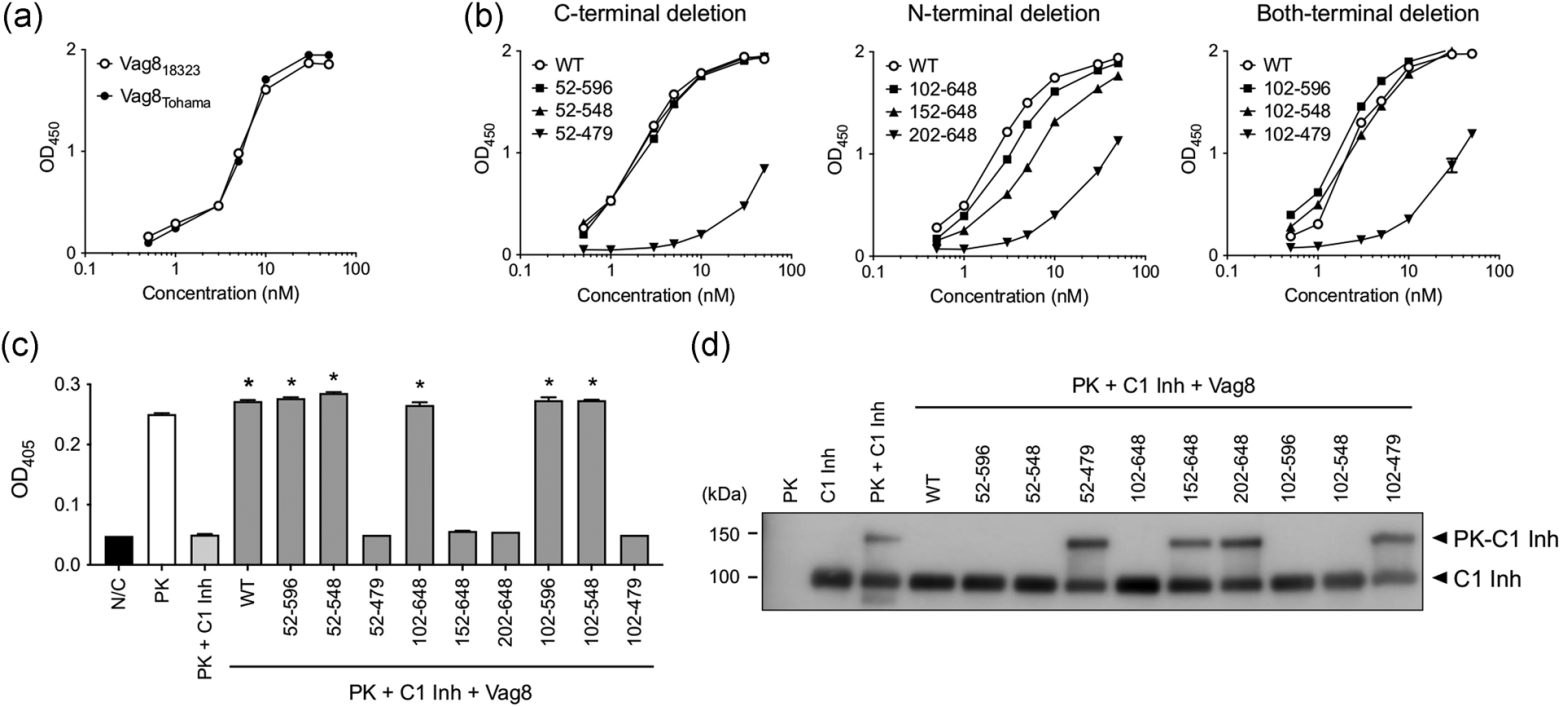
Abilities of Vag8 and its derivatives to interact with C1‐Inh. (a and b) Binding of Vag8_WT_ from *B. pertussis* 18323 (Vag8_18323_) and Tohama (Vag8_Tohama_), and truncated Vag8 derivatives to C1‐Inh analyzed by ELISA‐based binding assay. The data are presented as means (*n* = 3) and SEM. (c) Cancellation of the inhibitory effects of C1‐Inh on PK activity by truncated Vag8 derivatives. N/C is the negative control without PK, C1‐Inh, and Vag8s. The data are presented as means (*n* = 3) and SEM. Statistical analyses were carried out by one‐way analysis of variance and Tukey's multiple comparison test using Prism 8 (GraphPad Software). *P < .01. (d) Immunoblotting of C1‐Inh incubated with PK and truncated Vag8 derivatives. Note that C1‐Inh bound covalently to PK to form a complex with larger molecular mass (upper arrowhead)^10^

The results presented above indicate that the region of Vag8 consisting of amino‐acid residues 102–548 retains the ability to bind C1‐Inh and to block its inhibitory effect on PK. As neither Vag8_102‐479_ nor Vag8_202‐648_ showed the full activity of intact Vag8, the regions of amino‐acid residues 102–202 and 479–548 are likely required for the interaction with C1‐Inh, suggesting that at least two separate regions of Vag8 mediate the interaction with C1‐Inh. Alternatively, these two regions may stabilize the overall structure of Vag8, thus allowing it to interact with C1‐Inh. We found no characteristic motifs in Vag8_102‐548_ by public database BLAST (https://blast.ncbi.nlm.nih.gov/Blast.cgi) and Pfam (https://pfam.xfam.org) searches. The serine protease inhibitors, including C1‐Inh, contain the reactive center loop (RCL), which covalently binds the active site of target serine proteases as a “pseudo‐substrate” and inhibits their enzymatic activity.^10^ As the RCL was reported to be essential for Vag8 to interact with C1‐Inh,^11^ it may be important to localize the region of Vag8 required for interaction with the RCL to gain a further understanding of the molecular nature of the interaction between Vag8 and C1‐Inh.

## CONFLICT OF INTEREST

The authors declare that there are no conflicts of interest.
